# Explaining inequity in knowledge, attitude, and services related to HIV/AIDS: a systematic review

**DOI:** 10.1186/s12889-024-19329-5

**Published:** 2024-07-08

**Authors:** Aklilu Endalamaw, Charles F Gilks, Fentie Ambaw, Wondimeneh Shibabaw Shiferaw, Yibeltal Assefa

**Affiliations:** 1 School of Public Health, The University of Queensland, Brisbane, Australia; 2 College of Medicine and Health Sciences, Bahir Dar University, Bahir Dar, Ethiopia; 3 College of Medicine and Health Sciences, Debre Berhan University, Debre Berhan, Ethiopia

**Keywords:** Antiretroviral therapy, Attitude, Discrimination, Disparity, Equity, Equality, HIV testing, Knowledge, Stigma, Review

## Abstract

**Background:**

Equitable service provision and coverage are important responses to end the threat of the HIV/AIDS pandemic. Understanding inequity supports policies and programmes to deliver tailored interventions. There is continuous evidence generation on inequity in HIV/AIDS services. However, there was a lack of evidence on the global picture of inequity in behavioural and biomedical services related to HIV/AIDS. This systematic review assessed inequities in knowledge, attitude, HIV testing, and ART coverage across individual-level social groups and multiple (dis)advantage categories.

**Methods:**

This review followed the Preferred Reporting Items for Systematic Reviews and Meta-Analyses guideline, with a PROSPERO registration number CRD42024521247. The risk of bias was assessed by using Hoy et al’s and Joanna Brigg’s quality appraisal checklists for cross-sectional quantitative and qualitative studies, respectively. The search date was from inception to the final database search date (May 29, 2023). The included articles were either quantitative or qualitative studies. We used mixed-methods approach to analyse the data from the review articles. Quantitative descriptive analysis was conducted to estimate frequency of articles published from different countries around the world. Qualitative content analysis of the findings from the original studies was conducted using the PROGRESS plus framework which stands for: place of residence, occupation or employment status, gender, religion, education status, socioeconomic status, and social capital.

**Results:**

Out of 6,029 articles that were accessed and screened, only 72 articles met the inclusion criteria. More articles on HIV-related equity in knowledge, attitude, testing, and ART were published in developed countries than in developing countries. Individuals from higher-income households had better knowledge about HIV/AIDS. Unfavourable attitudes towards people living with HIV and HIV/AIDS-associated stigma were common among women. HIV/AIDS service coverage (HIV testing or ART coverage) was higher among richer and urban residents. HIV/AIDS-associated stigma and lower levels of knowledge about HIV/AIDS were observed among multiple disadvantageous groups due to the intersection of two or more identities.

**Conclusions:**

The current review revealed that there have been disparities in HIV/AIDS services between social classes. Ending service disparity towards the global threat of HIV/AIDS demands tailored interventions based on socially disadvantaged groups (e.g., poor, rural dwellers, and women) and intersectional determinants. There is a need to understand the deep-rooted causes of inequity and the challenges that an equity-oriented system faces over time. More studies on inequity are needed, including intersectional inequity, which has been rarely studied in developing countries.

**Supplementary Information:**

The online version contains supplementary material available at 10.1186/s12889-024-19329-5.

## Background

Comprehensive services are essential for an effective response to the HIV/AIDS epidemic. For instance, community members should have comparable knowledge about HIV/AIDS and accepting attitudes towards people living with HIV/AIDS. They should also have access to HIV testing and antiretroviral therapy (ART) [1]. These services will help countries achieve the three 95s: 95% of people living with HIV know their status, 95% of those who know their status will be on ART, and 95% of those on ART will achieve viral load suppression. This will enable countries to end the HIV/AIDS epidemic by 2030 as part of the sustainable development goal 3.3 [2–4].

Several efforts have been implemented to address inequity and ensure no one is left behind in the HIV/AIDS services. For instance, the ‘President’s Emergency Plan for AIDS Relief’ is being implemented for marginalised and underserved populations in many African countries [5–8]. Similarly, the ‘Federal Care and Prevention Project in the United States of America’ (USA) [9] and the ‘Centres for Disease Control and Prevention’ [10] have suggested holistic interventions to close disparities in social and living conditions. These interventions are supported by strategic collaborations between nations, non-governmental partners, the private and public sectors, effective leadership, global funds, and evidence-based practices based on disparities in social classes [11–15].

However, inequities and inequalities in HIV/AIDS services hinder the progress of the HIV/AIDS responses and universal health coverage (UHC) [16, 17]. HIV/AIDS-related morbidity, mortality, and disability-adjusted life years are higher in developing countries [18]. For instance, two-thirds of people living with HIV were in Africa in 2020 [19], where a few countries bore most of the burden [18]. The disparities largely affect individuals who have a lower probability of accessing services [20, 21]. In 2020, 19.3 million women and 16.7 million men were living with HIV worldwide [22]. Moreover, individuals under poor socioeconomic status and multiple disadvantageous identities had lower chance for accessing HIV/AIDS-related services [23–28]. Disadvantaged groups face inequalities because of their social category, which people form, transform, or maintain their identities. Due to its dynamic nature of being non-medical factors, they are influenced by the dynamic political situations [29–31], changing health policies [32], and emerging pandemic diseases [33–37]. Examples of groups and dynamics are racial and ethnic minorities, women, religious minorities, and other social groups, who influence their identities by their income. Multiple disadvantaged groups are those who cannot full-fill their health care needs due to their two or more disadvantaged classes [38]. In general, inequities in services are underscored in relation to social strata, which World Health Organization calls ‘social determinants of health’ [39].

Social determinants of health are the condition in which people are born, grow, work, and live and considered as non-medical factors that influence health outcomes. The ‘Commission on the Social Determinants of Health’ urged the marshalling of evidence on inequity for policy inputs [39]. This provides an understanding of disparities in the combined HIV/AIDS services to deliver tailored interventions [40]. Equity is a less investigated concept in the UHC period [41], and a priori systematic review focused on combination HIV prevention assessed empowerment, inclusion, and agency in low- and middle-income countries [42]. However, it did not address inequity in the HIV prevention services across social strata around the world. The current review aims to fill this gap.

This review aims to assess the inequities in knowledge about HIV/AIDS, attitudes towards people living with HIV or HIV-associated stigma, HIV testing practice, and ART coverage across different social classes. Specifically, it investigates which social strata demonstrates significant differences in knowledge, attitudes or stigma, and testing related to HIV/AIDS. Moreover, it explores how ART coverage varies among these social classes. Understanding the extent of these inequities is crucial for tailoring healthcare services based on social determinants of health. Additionally, this review will serve as a framework for future research by synthesising evidence from various sources, offering a comprehensive overview of existing knowledge. By examining global research on inequities, it sheds light on areas where equity-oriented research on HIV/AIDS services has been under investigated, particularly among specific social groups.

## Methods and materials

### Results reporting

This systematic review was conducted in accordance with the Preferred Reporting Items for Systematic Review and Meta-analysis guideline (PRISMA checklist) [43]. It helps to present a systematic, transparent, and complete methods and findings of the review. The protocol is registered in PROSPERO database with a registration number CRD42024521247.

### Eligibility criteria

Empirical articles published in English without a geographic limit were eligible. This systematic review was based on studies conducted in accordance with qualitative and quantitative methods approach. Those reported at least one of the selected outcome variables, including: knowledge about HIV/AIDS, accepting attitude towards people living with HIV, HIV associated stigma and discrimination, HIV testing, and ART coverage. Articles were included if they mentioned to assess the (in)equity, disparity, or (in)equality of those services based on one of the components of the PROGRESS Plus Framework, including multiple disadvantageous groups.

Articles on (in)equity that did not report HIV/AIDS services based on at least one component of PROGRESS plus, systematic reviews, meta-analyses, scoping review, any other types of review, conference abstracts, brief communications, letters to the editor, commentary, erratum, and retracted articles were excluded.

### Information sources

PubMed, Web of Science, Excerpta Medical Database (EMBASE), Scopus, and Google Scholar were searched. Reference lists of retrieved articles were also screened for additional article inquiry. Database search was conducted from the date of the first publication on the topic to September 15, 2022, and the search was updated on May 29, 2023. By the date of the first publication, we mean the earliest date when a relevant study on the topic was published. This means that we did not exclude any studies based on the year of publication. For example, according to the search strategy that we used in PubMed, the first study on the topic was published in 1984. Therefore, our systematic review process considered studies published from 1984 to the last search date (May 29, 2023).

### Search strategy

Search terms were knowledge, attitude, stigma, discrimination, test*, “HIV test”, **“**highly active antiretroviral therapy”, ART, HAART, antiretroviral, anti-retroviral, antiviral, therapy, “acquired immunodeficiency syndrome”, aids, hiv, “human immunodeficiency virus”, “HIV infections”, HIV/AIDS, “human immunodeficiency virus/acquired immunodeficiency syndrome”, equit*, inequit*, equalit*, equal, inequalit*, inequalit*, unequal, disparit*, and differenc*. The search strategy was constructed based on Boolean and truncation operators (AND, OR, *). An example search strategy used in EMBASE is (‘attitude’/exp OR ‘attitude’ OR ‘knowledge’/exp OR ‘knowledge’ OR ‘stigma’/exp OR ‘stigma’ OR ‘discrimination’/exp OR ‘discrimination’ OR ‘highly active antiretroviral therapy’/exp OR ‘highly active antiretroviral therapy’ OR ‘antiretroviral therapy’/exp OR ‘antiretroviral therapy’ OR ‘antiretrovirus agent’/exp OR ‘antiretrovirus agent’ OR ‘test’/exp OR test) AND (‘human immunodeficiency virus infection’/exp OR ‘human immunodeficiency virus’/exp OR ‘acquired immune deficiency syndrome’/exp OR ‘hiv aids’/exp) AND (‘equity’/exp OR ‘health disparity’/exp OR ‘diversity, equity and inclusion’/exp OR ‘inequality’/exp OR ‘disparity’/exp) AND [english]/lim. The full search strategy is found in the supplementary file (sT1).

### Selection process

One reviewer (AE) conducted a database search and screened based on their title and abstract. Subsequently, two reviewers (AE and WSS) discussed the eligibility criteria to ensure a shared understanding. Independently, they performed full-text screening. Afterward, they cross-checked the screened articles. Finally, through discussion, they resolved discrepancies between the two authors (AE and WSS).

### Data collection process

Qualitative and quantitative data were extracted and imported into Microsoft Excel^©^. Data included author(s)/year of publication, data collection period, country of publication, study design, statistical analysis, HIV/AIDS service category, study population, sample size, statistical analysis, and main findings. Main findings focus on differences between social classes. This include PROGRESS-plus refers to the place of residence, race/ethnicity/culture/language, occupation or employment status, gender/sex, religion, education, socioeconomic status, and social capital; ‘plus’ refers to personal characteristics (age) [44]. Intersectionality (multiple social identities) was also considered in this review [45]. Data from the quantitative studies were extracted based the significant level by evaluating the reported *p-*values and confidence intervals. If the required variables were reported as significantly associated social classes with no mentions of *p*-value or confidence intervals, we included them into the data set as per the report from the included studies. Regrading qualitative data, we reviewed the main findings content wise and extracted the reported findings to add into the data set.

### Data items

Mintzker et al. suggested PECO to establish research question for observational studies [46]. Accordingly, all people with no restriction were the population (P) for knowledge about HIV/AIDS, attitudes towards people living with HIV, and HIV testing, while people living with HIV is for stigma or discrimination and ART. Exposure (E) denotes variables in the PROGRESS-plus framework. Comparison (C) was the disadvantageous group in social classes (e.g., the rural category if urban is considered as exposure). The outcome (O) was inequality, inequity, or disparity in knowledge, attitude or stigma and discrimination, HIV testing, and ART coverage.

### Study risk of bias assessment

Hoy et al’s quality assessment criteria with a 10-point checklist was used to assess cross-sectional studies [47]. This checklist includes (1) target population representativeness, (2) true sampling frame, (3) random selection or census, (4) non-response bias handling, (5) data collected directly from the subjects, (6) case definition, (7) tool validity and reliability, (8) same mode of selection for all participants, (9) adequate length of study, and (10) appropriateness of numerator and denominator. The Joanna Brigg Institute’s (JBI) quality appraisal checklist was used for qualitative studies was used [48]. The JBI checklist includes (1) philosophical perspective and the research methodology congruity; (2) research methodology and the research question congruity; (3) congruity between the research methodology and the methods used to collect data; (4) research methodology and the representation and analysis of data congruity; (5) congruity between the research methodology and the interpretation of results; (6) a statement locating the researcher culturally or theoretically; (7) addressing researcher’s influence on the research and vice- versa; (8) participants, and their voices adequately represented; (9) evidence of ethical approval by an appropriate body; and (10) conclusions drawn in the research report flow from the analysis, or interpretation of the data. Two reviewers (AE and WSS) independently assessed the quality of the articles. Two reviewers solved disagreements raised during scoring articles for quality status by discussion.

### Synthesis methods

We used mixed-method analysis, including descriptive analysis (e.g., frequency of articles based on country) and qualitative content analysis for the main findings. The findings did not invite meta-analysis due to various analysis method and interest of outcome. Only a systematic review without meta-analysis was conducted. Both quantitative and qualitative findings were described as per the PROGRESS elements. Choropleth maps were generated by using Microsoft Excel to show available article distribution across the countries. The main findings were synthesised, framed, and interpreted based on the PROGRESS plus elements (place of residence, race/ethnicity/culture/language, occupation or employment status, gender/sex, religion, education status, socioeconomic status, and social capital, age [44] and intersectional identities (multiple social groups) [45].

## Results

### Study selection

**Fig. 1:**
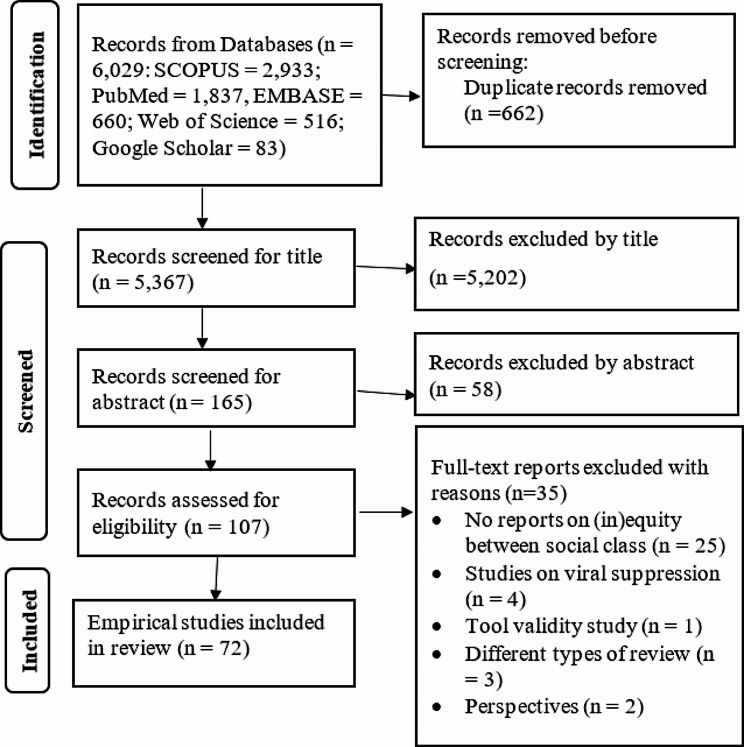
Article selection process

A total of 6,029 articles were identified and screened. Following the removal of duplicates as well as screening by reading title and abstract, 107 articles were selected for full-text review. Finally, 72 articles met the inclusion criteria and were included in the systematic review (Fig. 1).

### Study characteristics

**Fig. 2:**
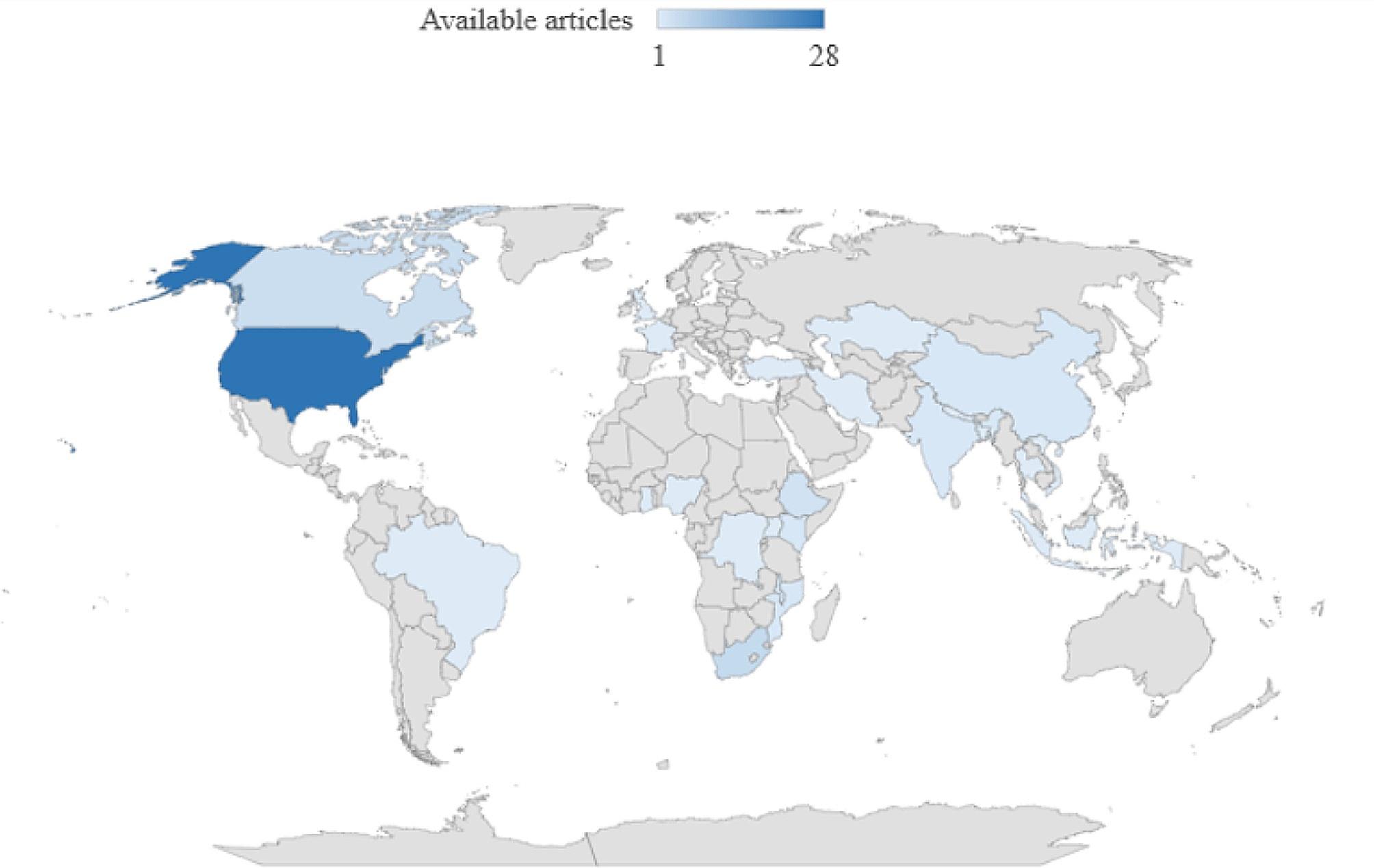
Available articles distribution across countries

HIV/AIDS service inequality was observed in low, lower-middle, upper-middle- and high-income countries. Based on study settings, 31 articles were from North America (28 articles from the USA [28, 49–75] and 3 articles from Canada [76–78]); 20 articles were from Africa (Ethiopia, Mozambique, Uganda, Ghana, Kenya, South Africa, Malawi, Nigeria, East Africa, sub-Saharan African countries) [26, 79–97], seven articles were from south east Asia (Nepal, Vietnam, Thailand, India, Indonesia, Bangladesh) [98–104], five articles were from the European region (France, UK, Turkey, Kazakhstan, 35 European countries) [105–109], three articles were from China [110–112], one article each from Latin America and Caribbean countries [113], low-and middle-income countries [114], Eastern Mediterranean (Iran) [115], African, Caribbean and southeast Asia countries [116], 49 countries [117], and Brazil [118]. Based on the frequency of publication by year, four articles were published before 2000, six articles were published between 2000 and 2005, six articles were published between 2006 and 2010, twenty articles were published between 2011 and 2015, twenty-three articles were published between 2016 and 2020, and thirteen articles were published after 2020 (Table 1). Figure 2 displays article distribution according to each country. To illustrate, 31 articles from North America are displayed on the map after classified to corresponding country, namely, 28 articles for the USA and 3 articles for Canada (Fig. 2).

**Table 1:** Characteristics of included articles

Author/Year	GJKJL	Study period	Study population	Sample size	Country	Analysis	PROGRESS Plus	Category
	Publication Year							
Agwu AL et al/2011	2011–2015	2002–2008	Youth and adults	3,127	United states of America (USA)	Regression	Age	ART coverage
Arifin H et al/2022	After 2020	2017	Female youths (15–24 years)	12,691	Indonesia	Regression	Residence, income, information	Attitude towards people living with HIV
Arnold M et al/2009	2006–2010	1996–2001	Population (all)	4,211	USA	Regression	Race/ethnicity	ART coverage
Asiedu GB et al/2014	2011–2015	2010	Adult with HIV	17	Ghana	Thematic analysis	Gender	Perceived Stigma
Astawesegn FH et al/2022	After 2020	2016	women (15–49 years)	45,476	East Africa	ECI and decomposition	Wealth	HIV test
Ataro Z et al/2020	2016–2020	2018	Adult with HIV	412	Ethiopia	Multiple linear regression	Gender	Perceived stigma
Atteraya M et al/2015	2016–2020	2011	women (15–49 years)	11,273	Nepal	Logistic regression	Caste & ethnicity	Knowledge
Behel SK et al/2008	2006–2010	1998–2000	MSM	2,424	USA	Logistic regression	Race/ethnicity	HIV test
Brown LK et al/1990	Before 2000	1987–1988	5th, 7th and tenth grade students	441	USA	ANOVA	Education	Knowledge and attitude
Burlew AK/2007	2006–2010	Not reported	Adult (> 18 years)	448	USA	Covariance test	Age	Knowledge
Chirawa 2019	2016–2020	2004, 2010 and 2016	Adults (all)	76,455	Malawi	ECI, Wagstaff decomposed ECI	Wealth	knowledge
Ebrahim SH et al/ 2004	2000–2005	2001	adults (18 to 64)	162, 962	USA	Logistic regression	Race/ethnicity	HIV test and knowledge
Elliott L et al/1992	Before 2000	1989–1990	adult male	733	United Kingdom (UK)	Descriptive (chi-square)	Majority and Minority ethnicity	knowledge and attitude
Faust L et al/2017	2016–2020	2013	adult (all)	56,307	Nigeria	Logistic regression	Sociodemographic & wealth	Knowledge
Fleishman JA et al/2012	2011–2015	2002–2008	Adult with HIV	14,092	USA	Logistic regression	Sex, race, age	ART coverage
Garofalo R et al/2015	2011–2015	2009	Young men who have sex with men	344	USA	ANOVA	Education and Race/ethnicity	Knowledge
Geary C et al/2014	2011–2015	2011	Adult with HIV	862	Ethiopia, Mozambique, Uganda	Logistic regression	Gender	Stigma
Gebo AK et al/2005	2000–2005	2001	Adult with HIV	10,905	USA	Logistic regression	Race, gender, age	ART coverage
Girum T et al/2018	2016–2020	2010–2016	Adult with HIV	399,000	Ethiopia	Difference (descriptive)	Gender	ART coverage
Gutiérrez JP & Trossero A. 2021	After 2020	2008–2018	Young female (15–24 years)	104,109	Latin America and Caribbean countries	CI	Wealth	HIV test and knowledge
Guwani JM et al/2004	2000–2005	1996	adult with HIV	865	USA	Logistic regression	Race/ethnicity	ART coverage
Hall HI et al/2013	2011–2015	2009	Adult with HIV	1,148,200	USA	Logistic regression	Sex, age, race/ethnicity	ART coverage
Hamidouche M et al/2022	After 2020	2010–2018	Adult	358, 591	18 sub-Saharan African countries	Relative and slope index of inequalities	Wealth	Knowledge, attitude and HIV test
Jaworsky D et al/2018	2016–2020	2015	Women with HIV	675	Canada	Linear regression	Geography	Stigma
Jesmin S.S. & Rahman M/2018	2016–2020	2004–2014	Women	11,428 in 2004 & 16,755 in 2014	Bangladesh	Logistic regression	Age, wealth, education, residence, employment	Knowledge
Kerrigan D et al/2017	2016–2020	2008–2009	Adult with HIV	900	Brazil	Logistic regression	Gender, race/ethnicity, education, religion, income	Stigma and discrimination
Landovitz RJ et al/2017	2016–2020	2010	Adult with HIV	9,566	USA	Logistic regression	Race/ethnicity	ART coverage
Lemly DC et al/2009	2006–2010	1998–2005	Adult with HIV	2,605	USA	Logistic regression	Race/ethnicity and Gender	ART coverage
Li X et al/2004	2000–2005	2000	College students	1,081	China	ANOVA	Resident, gender, education	Knowledge
Lo CC et al/2018	2016–2020	2013–2014	Adults	18,574	USA	Logistic regression	Age, gender, employment	HIV test
Loutfy MR et al/2012	2011–2015	2007–2009	Adult with HIV	1,026	Canada	Correlation	Gender, ethnicity	Stigma
McNaghten AD et al/2003	2000–2005	1998 and 1999	Adult with HIV	9,530	USA	Logistic regression	Gender, ethnicity	ART coverage
Metz VE et al/2017	2016–2020	2014 and 2015	Adults with opiod use disorder	138	USA	Descriptive (chi-square)	Race/ethnicity	Knowledge
Miller J E 2000	2000–2005	1998	Adult	460	USA	Descriptive (chi-square)	Education	Knowledge
Mori M et al/2015	2011–2015	2003–2013	children with HIV	2,101	South Africa	Multiple linear regression	Gender	ART coverage
Moyo S et al/2018	2016–2020	2007–2012	Adult with HIV	5,053	Kenya and South Africa	Logistic regression	Gender, employment, age, residence	ART coverage
Mudingayi A et al/2011	2011–2015	2005	Street on adolescent and youths	200	Democratic republic of Congo	Chi-square	Gender	Knowledge
Mugoya GCT et al/2014	2011–2015	2008–2009	Adults	11,818	Kenya	Logistic regression	Gender	Stigma
Ntata PRT et al/2008	2006–2010	2007	University students	314	Malawi	Logistic regression	Gender	Knowledge
Ojikutu B et al/2013	2011–2015	2010–2011	Black people	1,060	USA	Logistic regression	Birthplace	Knowledge, attitude and HIV test
Pannetier J et al/2016	2016–2020	2007	Adults with HIV	513	Thailand	Logistic regression	Gender	Stigma
Rapkin AJ&Erickson PI/990	Before 2000	Not reported	Adults	535	USA	ANOVA	Ethnicity	Knowledge
Rohleder P et al/2012	2011–2015	Not reported	disabled individuals	285	South Africa	Difference (descriptive)	Gender	Knowledge
Tas¸ci S et al/2008	2006–2010	2004–2005	University students	542	Turkey	Difference (descriptive)	Education	Knowledge
van Melle A et al/2015	2011–2015	2012	Adults	896	France	Logistic regression	Geography	Knowledge, attitude and HIV test
Waldner LK et al/1999	Before 2000	Not reported	University students	190	USA	ANOVA	Race/ethnicity	Knowledge
Yang F et al/2021	After 2020	2003–2018	Young women	282,757	low-and middle-income countries	Difference (percentage point)	Residence, income, Education	knowledge and trend of disparity
Yao J et al/2014	2011–2015	2006–2009	Adults	1,025	Mozambique	Spatial, descriptive, logistic regression	Geography, education, household possession index	HIV test
Zhan J et al/2021	After 2020	2019	University students	10,665	China	Logistic regression	Knowledge, education	HIV test
Zhang S et al/2014	2011–2015	2005–2007	Medicaid Population HIV positive	32,513	USA	Logistic regression	Age, residence, race/ethnicity	ART coverage
Zhang S et al/2013	2011–2015	2005–2007	Medicaid enrolees HIV positive pregnant women	3,259	USA	Logistic regression	Race/ethnicity	ART coverage
Zhussupov B et al/2014	2011–2015	2007	Migrant workers	422	Kazakhstan	Logistic regression	Gender	Knowledge
Larose A et al/2011	2011–2015	2002–2003	Adults (18–49 years)	106,705	49 countries	Logistic regression	Wealth	HIV test
Wabir N et al/2013	2011–2015	2008	Adults (15–64 years)	10,856	South Africa	Logistic regression	Income	HIV test and knowledge
Ngandu NK et al/2017	2016–2020	2012	pregnant women	8,618	South Africa	CI and decomposition of CI	Wealth	HIV test
Kim SW et al/2016	2016–2020	2004–2010	Adults (15–54 years)	44,401	Malawi	CI and decomposition of CI	Wealth	HIV test
Chu DT et al/2019	2016–2020	2014	Pregnant women 915 − 49 years)	1,484	Vietnam	CI, concentration curve and logistic regression	Age, ethnicity, education, residence, wealth	HIV test
Ante-Testard PA et al/2020	2016–2020	2003–2016	Adults (15–59 years)	537,784	sub-Saharan Africa countries	Relative and absolute index inequality, meta-analysis, trend inequality	Age, wealth, marital status, education	HIV test
McClarty LM et al/2021	After 2020	2017	Adults (18–69 years)	703	Canada	Equiplot	Age, sex, ethnicity, geography, immigration status, exposure status	ART coverage
Laut K et al/2018	2016–2020	2004–2015	Adults	23,043	35 European countries	Logistic regression with a generalised equation model	Geography (between region or country) and time difference (2004–2005, 2009–2010, 2014–2015)	ART coverage
Auld AF et al/2015	2011–2015	2002–2013	Adults	765,087	African, Caribbean and Southeast Asia countries	Ratio	Gender	ART coverage
Beer L et al/2016	2016–2020	2009–2013	Adults	22,081	USA	Regression-based difference-in-difference approach	Race/ethnicity	ART coverage
Sharma SK et al. 2022	After 2020	2015/2016	Pregnant women (15–49 years)	122,351	India	CI, logistic regression, Wagstaff decomposition of CI	Wealth	HIV test
Chipanta D et al/ 2022	After 2020	2015–2018	People living with HIV (men, women and adolescent)	444 to 3,199	sub-Saharan Africa countries	Concentration curves, computed concentration indices	Wealth and contributors	HIV test and ART coverage (first and second 90)
McCree DH et al/2023	After 2020	2018–2019	People living with HIV (adults)	3850	USA	Prevalence ratios	Sexual orientation, race/ethnicity, income, or social class, and/or injection drug use	HIV associated discrimination
Chakrapani V et al/2023	After 2020	2019	People living with HIV	Four focus group discussion	India	Coding and categorising	Gender, HIV status, sex worker status	ART initiation
Ghasemi E et al/2021	After 2020	2018–2020	Immigrants	25	Iran	Thematic analysis	Migration, gender, and drug addiction	Stigma
Barrington C et al/2021	After 2020	Not mentioned	Gay Latino men living with HIV	14	USA	Thematic analysis	Being gay, Latino, undocumented) and intersecting structures (healthcare, immigration policy, institutionalised homophobia	HIV care continuum
Algarin AB et al/2019	2016–2020	2014 to 2017	People living with HIV	932	USA	Multinomial regression	Age, sex, race, ethnicity and sexual orientation	Stigma
Agénor M et al/2019	2016–2020	2013–2017	Adult men and women	60,867 women and 52,201 men	USA	Logistic regression	Sexual orientation identity, race/ethnicity	Lifetime HIV testing
Rountree MA et al/2016	2016–2020	Not mentioned	Women (26 to 40 years)	25	USA	Qualitative analysis	Ethnicity and language	HIV knowledge
Konkor I et al/2020	2016–2020	Not mentioned	Men	155	Canada	Negative log-log link function	Sexual orientation and ethnicity	HIV testing

ART: Antiretroviral Therapy; CC: concentration curve; CI: concentration index; ECI: Erreygers’ Concentration Index; HIV: Human Immunodeficiency Virus; NA: Not applicable; UK: United Kingdom; USA: the United States of America

### Risk of bias in studies

**Fig. 3:**
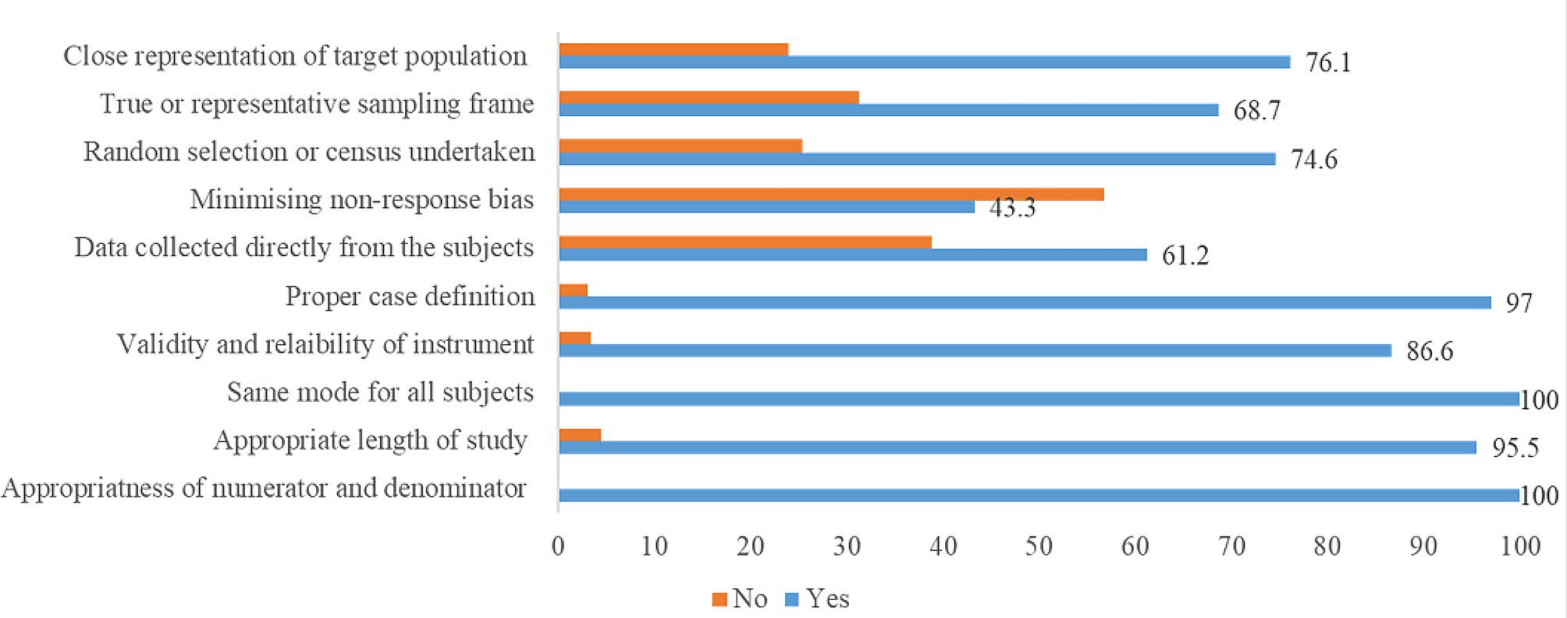
Percentage distribution of articles quality per quality indicators

Out of 72 articles assessed for risk of bias, 67 were quantitative and 5 were qualitative articles. Of 67 quantitative articles, 48 articles were found low and 19 have moderate risk for bias (Fig. 3).

Five qualitative studies were assessed using the JBI’s quality appraisal criteria for qualitative studies. Four have scored 8 out of 10 because they missed a statement ‘locating the research culturally or theoretically’ and ‘addressing the researcher’s influence on the research and vice versa’. One article has scored 9 out of 10 because it missed a statement ‘locating the research culturally or theoretically’. The detailed quality appraisal checklist with articles is available in the supplementary file (sT2).

## Results of synthesis

### Knowledge about HIV/AIDS

Rural resident [100, 110, 114], unemployed [100], women [108, 110, 119], traditional religious followers [83], lower education status [52, 65, 83, 100, 110, 114, 120], lower income status [23, 26, 82, 83, 86, 100, 114], and non-US born people compared to US-born people [66] had lower knowledge about HIV/AIDS. One, in the United States [28], assessed intersectionality inequity (being monolingual and Latino) concluded that monolingual Latina women had a lower level of HIV/AIDS knowledge than their counterparts. Whites than Blacks or Latinos [68, 120], high-caste group than low-caste group in Nepal [99], and English speakers than Spanish speakers in the USA [67] were more knowlegeable about HIV/AIDS. Regarding age category, there were two studies with contradictory findings [53, 83]. One study each reported that there was no ethnic-based disparity in knowledge about HIV/AIDS among opioid users [64], and there was no gender based disparity in Democratic Republic of the Congo [89] and Malawi [91].

### Attitude towards people living with HIV

One study revealed that a rural resident among female youths had exhibited a better attitude towards people living with HIV [98]. However, others, non-US born people [66], Asian men compared to an ethnic majority group in UK [121], individuals with lower education status [52] and lower income status [86], had showed a lower accepting attitude towards people living with HIV. In contrast, one study reported that individuals with middle-to-richest group had lower accepting attitude towards people living with HIV compared to the poorest [98].

### HIV associated stigma

Compared to their counterparts, northern and rural regions of Ontario [76], black women and Asian/Latin American/unspecified men [111], women [79, 81, 84, 90, 101], and younger [118] were more likely to experience HIV-associated stigma. Similarly, individuals with multiple disadvantaged identities, such as immigrants, females, and drug users [122], African Caribbean and black men [78], sexual orientation, race/ethnicity, income, social class, and injection [73], and non-white Latinos [75] also faced a higher rate of HIV-associated stigma and discrimination. This phenomenon is known as intersectional inequity, which means that different social identities and systems of oppression interact and create unique experiences of marginalization.

### HIV testing

Compared to their counterparts, rural residents [102], non-US born people [66], Whites compared to Blacks or Latinos [54], people from ethnic groups [102], lower education status [93, 102], and lower income status [23, 26, 80, 86, 93, 94, 103, 117, 123] had less access to HIV testing. However, one study revealed that pregnant women with lower income status had more access to HIV testing in South Africa [97]. Similarly, younger [102], women in the USA and sub-Saharan African countries [62, 123], employed [62], remote villages on the Maroni compared to Coastal areas in France [107] had more access to HIV testing. Bisexual women and gay men had a higher lifetime HIV testing rate than their heterosexual counterparts [72]. Finally, a study in the USA showed that there was no ethnic-based disparity in HIV testing among men who performed sex with men [51].

### ART coverage

Rural residents [88], transgender women compared to females [118], lower education status [118], lower income status [96, 118], younger [49, 57, 77, 88], Black/ Hispanic/ Latino/ non-white people [50, 57–61, 69, 70, 77, 118, 124, 125], southern Europe than western Europe [109] had a lower access to ART compared to their counterparts. However, regarding gender, there was contradiction: six studies reported that women had less access to ART [55, 57, 59, 61, 87, 125], while one study reported that women in ten African countries, Haiti and Vietnam [116] had more access to ART. Additionally, employed individual had better access to ART [88]. On the other hand, multiple disadvantaged groups, such as HIV-infected or a sex worker and a lack of gender affirmation (e.g., misgendering) [104] had less access to ART. One study each reported that there was no racial- [71] and gender-based [126] difference in ART coverage. Undocumented participants linked their immigration status to their ability to get work, which then affected their HIV care [74].

## Discussion

Evidence shows that HIV/AIDS service coverage varies according to place of residence, race or ethnicity, employment status or occupation, gender, religion, education, and socioeconomic status because of disparities in living and working conditions. These disparities can influence people’s knowledge, attitudes, and behaviours related to HIV/AIDS, as well as their uptake of HIV/AIDS services [127]. In settings characterised by significant resource scarcity, low-income nations, low gross domestic product, conflict areas, high corruption, and high gender inequality, there may be significant disparities in HIV/AIDS services and related to knowledge, attitude, stigma, and discrimination [128]. While most studies report that poorer people and women have lower access, some studies have found contradictions, indicating that poorer people and women actually had greater access to certain services, such as HIV testing [97]. There are discrepancies among the included studies because of study period, population, sample size, analysis, and context. This may indicate the application of different approaches and research in public health across different study periods.

Community engagement, inclusiveness, and collaborative response have become the priority strategies in health policy and care delivery. These strategies aim to address the social determinants of health and reduce health inequities. To provide comprehensive action on determinants, primary health care needs multisectoral action and policy, public and clinical care, and community engagement [129]. Legal and structural priorities are also frequently emphasised in cross-cutting social and environmental contexts [130]. The behavioural-based interventional and implementational research aims to narrow the gap of the social determinants-based inequality in HIV/AIDS services, using evidence from previous studies that show how factors such as income, education, and gender affect access to prevention, testing, and treatment [131, 132]. These types of studies need to accommodate evidence that shows different patterns of disparity in HIV/AIDS services among age groups [53, 83, 102, 118]. On the other hand, there has been historical, social, cultural, and economic consistency in ramping up gender-based inequity in HIV/AIDS services: as evidenced by higher HIV-associated stigma [79, 81, 84, 90, 101], lower knowledge about HIV/AIDS [108, 110, 119] and lower ART coverage [55, 57, 59, 61, 87, 125] among women. It is important to note that HIV/AIDS services are interconnected. Therefore, higher stigma among women affects their health-seeking behaviour and their access to other HIV/AIDS services [133]. It is thus true that the UN approved the need for global calls for gender equality, which enacted measures to engage women in political positions, empower them in the community, provide better opportunities for education, implement affirmative action in employment, and increase the number of female health workers in health sectors [134]. These strategies may close gender inequity in a country where women have fewer opportunities to learn and earn a living or a high gender pay gap [135, 136]. However, more research is needed to evaluate their effectiveness and impact on HIV/AIDS outcomes.

Studies reported that rural residents had lower knowledge about HIV/AIDS [100, 110, 114], HIV testing [102], and ART coverage [88]. This evidence supports the necessity of global and national strategies to reach marginalised and hard-to-reach populations, which usually reside in rural settings [137]. Primary health care, one of the long-term strategies to reach the rural population, aims to expand walk-in health clinics [138]. This will help in distributing health resources and infrastructure to rural areas so that they can have equal access to health information, advice, and education. Thus, the vicious cycle of HIV/AIDS services might be maintained in rural settings. The vicious cycle of HIV/AIDS services refers to the phenomenon that people who have more knowledge about HIV/AIDS are more likely to access prevention, testing, and treatment services, while those who have less knowledge are more likely to miss out on these services and remain at risk of infection or poor health outcomes [139]. However, the reverse may be true in that people who are tested for HIV or linked to an ART clinic may develop comprehensive knowledge about HIV/AIDS even though they have no prior information [140]. Moreover, even though rural residents have lower knowledge about HIV/AIDS, one study discovered female youths in rural areas had a better accepting attitude towards people living with HIV [98]. Therefore, community health workers could have a positive impact on narrowing the gap between urban and rural areas, aiming to maximise service coverage in rural areas and maintain the optimum level in urban areas [141].

Disparities in HIV/AIDS services are observed between ethnic groups; lower ART coverage [50, 57–61, 69, 70, 77, 118, 124, 125], knowledge about HIV/AIDS [54, 68, 99, 120], attitudes towards people living with HIV [111, 121], and HIV test coverage [102] were among ethnic minority groups. As most studies report, there is greater inequity between ethnic minority and majority groups. However, minority groups are inaccessible to many healthcare services because lower education and economic conditions have seriously affected them [142]. It is imperative to consider a further breakdown of social classes beyond ethnicity. For instance, the migrant populations face several challenges in accessing HIV/AIDS-related services, including individual- and organizational-level barriers [143]. Therefore, ‘culturally-tailored’ [144], ‘eHealth and mHealth’ [145], and ‘community-coalition-driven’ [146] interventions brought important change to health knowledge and self-management, psychosocial outcomes among ethnic minority and historically underserved populations.

Inequality in HIV/AIDS services is also apparent between household wealth rank, or income, occupation, and education status. Most studies reported that people with lower income had lower knowledge about HIV/AIDS [23, 26, 82, 83, 86, 100, 114] and HIV test coverage [23, 26, 80, 86, 93, 94, 103, 117, 123]. Similarly, people with lower education status had lower access to HIV/AIDS services [52, 65, 83, 93, 100, 102, 110, 112, 114, 118, 120]. Unless care consideration made for non-illitrate, literacy had been identified as one of the root causes of disparity, including in developed nations [147]. There is a good opportunity that, gradually, the number of literate people is increasing over time, including in developing countries [148]. This may create a suitable environment to prepare, conduct, and disseminate health literacy. Moreover, there should be an emphasis on quality education to close the gap between educated and uneducated people in knowledge about HIV/AIDS, attitude towards people living with HIV or HIV associated stigma and discrimination, and HIV service uptake.

Interventions for narrowing the gap in education-based disparity through increasing the education status of people with lower education status will have a positive impact on socioeconomic inequality. This will happen because differences in education status result in socioeconomic inequality in HIV testing [80, 94, 103] and knowledge about HIV/AIDS [82]. However, in most cases, individuals with a lower household wealth index had less knowledge about HIV/AIDS [23, 26, 83, 86, 100, 114] and accepting attitude towards people living with HIV [86], and less access to HIV testing [23, 26, 80, 86, 93, 94, 103, 117, 123] and ART [118]. Therefore, ongoing interventions are required to economically empower the poorest and groups of the population living in poverty that increase health-seeking behaviour and the ability to pay for health care expenditure, which can support people in multiple disadvantages identity [149].

Multiple disadvantages identity is called intersectional identities, which were a determinant of HIV/AIDS-related services, similar to other health care practises [150, 151]. Different axes of intersectionality prevent women from accessing HIV care [152]. Effective strategies are needed to address them. For example, implementation study in Ghana revealed that peer support reduced intersectional stigma [153].

Overall, the included studies were varied in year of study, contexts, study population, methodology, and outcome of interest. Rare studies have investigated patterns of inequity based on religion, social capital, disability, and language, and more research is needed on these social determinants. The contributors to socioeconomic inequalities and the extent of inequality among multiple disadvantageous groups over time were not well explored. The World Health Organisation launched the building blocks of the health system, which play a great role on equity [154]. Therefore, assessing inequities from this perspective would make it practical to address identified barriers comprehensively.

### Strength and limitations

This is the first systematic review to assess inequities in knowledge of HIV/AIDS, attitude towards people living with HIV or HIV associated stigma, HIV testing, and ART coverage. Despite this, it has some limitations. First, this review included only articles published in English, and there may be other non-English articles with supporting or contradicting evidence. Second, this review included research with wide variability in methods, making it difficult to quantify inequity using meta-analysis. Third, this review included prior research if it aimed to or mentioned (in)equity or (in)equality, which in some instances may be social determinants assessed differently but are not part of this review.

## Conclusions

To conclude, younger, uneducated, individuals with poor household income, the unemployed, rural residents, and ethnic minorities seem to have benefited from HIV/AIDS services less than their counterparts. HIV/AIDS service inequality was unlimited based on a lower or higher HIV prevalence rate. It means there was inequality in HIV/AIDS services in both countries, with lower and higher HIV burdens. Individuals who live under two or more socially disadvantaged conditions were deprived of HIV-associated knowledge and other services in developing countries that underscored more evidence is needed on intersectionality in developing countries. Ending service disparity and thus the global threat of HIV/AIDS demands multifaceted tailored interventions. Additionally, inequality-aimed research on HIV/AIDS services was more researched in developed countries where a relatively lower HIV prevalence than in high HIV burden countries. There is also a need to understand the deep-rooted causes of inequity and the challenges that an equity-oriented system faces over time.

### Electronic supplementary material

Below is the link to the electronic supplementary material.

**Supplementary Material 1: sT1:** Search strategy

**Supplementary Material 2: sT2:** Risk of bias assessment of included articles

## Data Availability

The required data is available in the manuscript and its supplementary file.

## Abbreviations

ART: Antiretroviral therapy
UHC: Universal Health Coverage
UK: United Kingdom
USA: United States of America

## References

[1] Granich R, Crowley S, Vitoria M, Smyth C, Kahn JG, Bennett R (2010). Highly active antiretroviral treatment as prevention of HIV transmission: review of scientific evidence and update. Curr Opin HIV AIDS.

[2] Frescura L, Godfrey-Faussett P, Feizzadeh AA, El-Sadr W, Syarif O, Ghys PD (2022). Achieving the 95 95 95 targets for all: a pathway to ending AIDS. PLoS ONE.

[3] Doyle MW, Stiglitz JE (2014). Eliminating extreme inequality: a sustainable development goal, 2015–2030. Ethics Int Affairs.

[4] UNAIDS. The Sustainable Development Goals and the HIV response. Geneva, Switzerland: Joint United Nations Programme on HIV/AIDS.

[5] Odekunle FF, Odekunle RO. The impact of the US president’s emergency plan for AIDS relief (PEPFAR) HIV and AIDS program on the Nigerian health system. Pan Afr Med J. 2016;25. 10.11604/pamj.2016.25.143.9987 PMC5326074 28292105

[6] Health–Americas TLR. Leaving no one behind: a call to increase access to HIV care for marginalised communities in the Americas. Lancet Reg Health-Americas. 2021;4. 10.1016/j.lana.2021.100155 PMC9903880 36776709

[7] Cornish F, Priego-Hernandez J, Campbell C, Mburu G, McLean S (2014). The impact of community mobilisation on HIV prevention in middle and low income countries: a systematic review and critique. AIDS Behav.

[8] Mukherjee A, Das M (2011). Mainstreaming gender in HIV programs: issues, challenges and way forward. Eastern J Med.

[9] De Jesus M, Williams DR (2018). The care and prevention in the United States demonstration project: a call for more focus on the social determinants of HIV/AIDS. Public Health Rep.

[10] Centres for Disease Control and Prevention. Establishing a holistic framework to reduce inequities in HIV, viral hepatitis, STDs, and tuberculosis in the United States USA: Centres for Disease Control and prevention; 2010 [ https://npin.cdc.gov/publication/establishing-holistic-framework-reduce-inequities-hiv-viral-hepatitis-stds-and

[11] NIH Strategic Plan for HIV and HIV-National Institute of Health: the Office of AIDS Research, Related Research. FY 2021–2025 [ https://www.oar.nih.gov/about/directors-corner/letters-director-fiscal-year-2021-2025-nih-strategic-plan-hiv-and-hiv-related-research

[12] World Health Organization. Global health sector strategy on HIV 2016–2021. Towards ending AIDS. World Health Organization; 2016.

[13] Jones DS, Tshimanga M, Woelk G, Nsubuga P, Sunderland NL, Hader SL (2009). Increasing leadership capacity for HIV/AIDS programmes by strengthening public health epidemiology and management training in Zimbabwe. Hum Resour Health.

[14] UNAIDS. The ‘Education plus’ initiative (2021–2025) - empowerment of adolescent girls and young women in sub-Saharan Africa: UNAIDS; [ https://www.unaids.org/en/topics/education-plus

[15] Braveman P, Egerter S, Williams DR (2011). The social determinants of health: coming of age. Annu Rev Public Health.

[16] Endalamaw A, Gilks CF, Ambaw F, Assefa Y (2022). Universality of universal health coverage: a scoping review. PLoS ONE.

[17] Inequalities are blocking the (2022). End of the AIDS pandemic [press release].

[18] Gona PN, Gona CM, Ballout S, Rao SR, Kimokoti R, Mapoma CC (2020). Burden and changes in HIV/AIDS morbidity and mortality in Southern Africa Development Community Countries, 1990–2017. BMC Public Health.

[19] World Health Organization. HIV/AIDS: Key facts 2021 [ https://www.who.int/news-room/fact-sheets/detail/hiv-aids#:~:text=There were an estimated 37.7,2.0 million%5D people acquired HIV

[20] Tesfayohannes S, Shine S, Mekuria A, Moges S. Mortality and its predictors among Adult Human Immune-Deficiency Virus-infected patients attending their antiretroviral treatment at Health Centers, Addis Ababa, Ethiopia: Multicenter Retrospective Cohort Study. AIDS Research & Treatment; 2022. 10.1155/2022/6128718 PMC9512605 36172060

[21] Leshargie CT, Demant D, Burrowes S, Frawley J (2022). Incidence and predictors of mortality among adolescents on antiretroviral therapy in Amhara Region, Ethiopia: a retrospective cohort analysis. BMJ open.

[22] UNAIDS. GLOBAL AND REGIONAL DATA (2021). Building on two decades of progress against AIDS.

[23] Gutierrez JP, Trossero A. Socioeconomic inequalities in HIV knowledge, HIV testing, and condom use among adolescent and young women in Latin America and the Caribbean. Revista Panamericana De Salud Publica-Pan American. J Public Health. 2021;45. 10.26633/RPSP.2021.47 PMC8147735 34054931

[24] Chirwa GC (2020). Who knows more, and why? Explaining socioeconomic-related inequality in knowledge about HIV in Malawi. Sci Afr.

[25] Chirwa GC, Sithole L, Jamu E (2019). Socio-economic inequality in Comprehensive Knowledge about HIV in Malawi. Malawi Med Journal: J Med Association Malawi.

[26] Wabiri N, Taffa N (2013). Socio-economic inequality and HIV in South Africa. BMC Public Health.

[27] Kim SW, Skordis-Worrall J, Haghparast-Bidgoli H, Pulkki-Brännström AM (2016). Socio-economic inequity in HIV testing in Malawi. Glob Health Action.

[28] Rountree MA, Granillo T, Bagwell-Gray M (2016). Promotion of Latina health: intersectionality of IPV and risk for HIV/AIDS. Violence against Women.

[29] Lavers T (2019). Towards universal health coverage in Ethiopia’s ‘developmental state’? The political drivers of health insurance. Soc Sci Med.

[30] Croke K (2020). The origins of Ethiopia’s primary health care expansion: the politics of state building and health system strengthening. Health Policy Plann.

[31] Østebø MT, Cogburn MD, Mandani AS (2018). The silencing of political context in health research in Ethiopia: why it should be a concern. Health Policy Plann.

[32] Gender. and COVID-19 Project and women in global health. Strengthen gender mainstreaming in WHO´s pandemic preparedness and response, Policy Brief. 2020.

[33] Moynihan R, Sanders S, Michaleff ZA, Scott AM, Clark J, To EJ (2021). Impact of COVID-19 pandemic on utilisation of healthcare services: a systematic review. BMJ open.

[34] Magnani RJ, Wirawan DN, Sawitri AAS, Mahendra I, Susanti D, Utami Ds NKAD (2022). The short-term effects of COVID-19 on HIV and AIDS control efforts among female sex workers in Indonesia. BMC Womens Health.

[35] Shi L, Tang W, Hu H, Qiu T, Marley G, Liu X (2021). The impact of COVID-19 pandemic on HIV care continuum in Jiangsu, China. BMC Infect Dis.

[36] Adugna A, Azanaw J, Melaku MS (2021). The Effect of COVID-19 on routine HIV Care services from Health Facilities in Northwest Ethiopia. HIV/AIDS (Auckland NZ).

[37] Varshney K, Ghosh P, Stiles H, Iriowen R (2022). Risk factors for COVID-19 mortality among people living with HIV: a scoping review. AIDS Behav.

[38] Nyamande FN, Mosquera PA, San Sebastián M, Gustafsson PE (2020). Intersectional equity in health care: assessing complex inequities in primary and secondary care utilization by gender and education in northern Sweden. Int J Equity Health.

[39] World Health Organization. Closing the gap in a generation: health equity through action on the social determinants of health. 2008. 10.1016/S0140-6736(08)61690-6 18994664

[40] Global, UNAIDS (2021). AIDS strategy 2021–2026: end inequalities.

[41] Endalamaw A, Gilks CF, Ambaw F, Habtewold TD, Assefa Y (2022). Universal Health Coverage for Antiretroviral Treatment: a review. Infect Dis Rep.

[42] Brody C, Sok S, Tuot S, Pantelic M, Restoy E, Yi S (2019). Do combination HIV prevention programmes result in increased empowerment, inclusion and agency to demand equal rights for marginalised populations in low-income and middle-income countries? A systematic review. BMJ Global Health.

[43] Page MJMJ, Bossuyt PM, Boutron I, Hoffmann TC, Mulrow CD (2021). The PRISMA 2020 statement: an updated guideline for reporting systematic reviews. BMJ.

[44] Cochrane Mehtods Equity. PROGRESS-Plus: the Cochrane collaboration; [cited 2022 September 30]. https://methods.cochrane.org/equity/projects/evidence-equity/progress-plus

[45] Bowleg L, Teti M, Malebranche DJ, Tschann JM (2013). It’s an uphill battle everyday: intersectionality, low-income black heterosexual men, and implications for HIV prevention research and interventions. Psychol men Masculinity.

[46] Mintzker Y, Blum D, Adler L. Replacing PICO in non-interventional studies. BMJ Evidence-Based Med. 2022. 10.1136/bmjebm-2021-111889 35017173

[47] Hoy D, Brooks P, Woolf A, Blyth F, March L, Bain C (2012). Assessing risk of bias in prevalence studies: modification of an existing tool and evidence of interrater agreement. J Clin Epidemiol.

[48] Lockwood C, Munn Z, Porritt K (2015). Qualitative research synthesis: methodological guidance for systematic reviewers utilizing meta-aggregation. JBI Evid Implement.

[49] Agwu AL, Fleishman JA, Korthuis PT, Siberry GK, Ellen JM, Gaur AH (2011). Disparities in antiretroviral treatment: a comparison of behaviorally HIV-infected youth and adults in the HIV Research Network. J Acquir Immune Defic Syndr.

[50] Arnold M, Hsu L, Pipkin S, McFarland W, Rutherford GW (2009). Race, place and AIDS: the role of socioeconomic context on racial disparities in treatment and survival in San Francisco. Soc Sci Med.

[51] Behel SK, MacKellar DA, Valleroy LA, Secura GM, Bingham T, Celentano DD (2008). HIV prevention services received at health care and HIV test providers by young men who have sex with men: an examination of racial disparities. J Urban Health.

[52] Brown LK, Nassau JH, Barone VJ (1990). Differences in AIDS knowledge and attitudes by grade level. J Sch Health.

[53] Burlew AK (2007). Age differences in knowledge about HIV transmission among African-American men and women. Psychol Rep.

[54] Ebrahim SH, Anderson JE, Weidle P, Purcell DW (2004). Race/ethnic disparities in HIV testing and knowledge about treatment for HIV/AIDS: United States, 2001. AIDS Patient Care STDS.

[55] Fleishman JA, Yehia BR, Moore RD, Gebo KA, Agwu AL (2012). Disparities in receipt of antiretroviral therapy among HIV-infected adults (2002–2008). Med Care.

[56] Garofalo R, Gayles T, Bottone PD, Ryan D, Kuhns LM, Mustanski B (2015). Racial/ethnic differences in HIV-related knowledge among young men who have sex with men and their association with condom errors. Health Educ J.

[57] Gebo KA, Fleishman JA, Conviser R, Reilly ED, Korthuis PT, Moore RD (2005). Racial and gender disparities in receipt of highly active antiretroviral therapy persist in a multistate sample of HIV patients in 2001. J Acquir Immune Defic Syndr.

[58] Guwani JM, Weech-Maldonado R (2004). Medicaid Managed Care and racial disparities in AIDS Treatment. Health Care Financ Rev.

[59] Hall HI, Frazier EL, Rhodes P, Holtgrave DR, Furlow-Parmley C, Tang T (2013). Differences in human immunodeficiency virus care and treatment among subpopulations in the United States. JAMA Intern Med.

[60] Landovitz RJ, Desmond KA, Leibowitz AA (2017). Antiretroviral therapy: racial disparities among publicly insured californians with HIV. J Health Care Poor Underserved.

[61] Lemly DC, Shepherd BE, Hulgan T, Rebeiro P, Stinnette S, Blackwell RB (2009). Race and sex differences in antiretroviral therapy use and mortality among HIV-infected persons in care. J Infect Dis.

[62] Lo CC, Runnels RC, Cheng TC (2018). Racial/ethnic differences in HIV testing: an application of the health services utilization model. SAGE Open Med.

[63] McNaghten AD, Hanson DL, Dworkin MS, Jones JL (2003). Differences in prescription of antiretroviral therapy in a large cohort of HIV-infected patients. J Acquir Immune Defic Syndr.

[64] Metz VE, Sullivan MA, Jones JD, Evans E, Luba R, Vogelman J (2017). Racial differences in HIV and HCV Risk behaviors, Transmission, and Prevention Knowledge among Non-treatment-seeking individuals with opioid Use Disorder. J Psychoact Drugs.

[65] Miller JE (2000). Differences in AIDS knowledge among Spanish and English speakers by socioeconomic status and ability to speak English. J Urban Health.

[66] Ojikutu B, Nnaji C, Sithole J, Schneider KL, Higgins-Biddle M, Cranston K (2013). All black people are not alike: differences in HIV testing patterns, knowledge, and experience of stigma between U.S.-born and non-U.S.-born blacks in Massachusetts. AIDS Patient Care STDS.

[67] Rapkin AJ, Erickson PI (1990). DIFFERENCES IN KNOWLEDGE OF AND RISK-FACTORS FOR AIDS BETWEEN HISPANIC AND NON-HISPANIC WOMEN ATTENDING AN URBAN FAMILY-PLANNING CLINIC. Aids.

[68] Waldner LK, Sikka A, Baig S (1999). Ethnicity and sex differences in university students’ knowledge of AIDS, fear of AIDS, and homophobia. J Homosex.

[69] Zhang S, McGoy SL, Dawes D, Fransua M, Rust G, Satcher D (2014). The potential for elimination of racial-ethnic disparities in HIV treatment initiation in the Medicaid population among 14 southern states. PLoS ONE.

[70] Zhang S, Senteio C, Felizzola J, Rust G (2013). Racial/ethnic disparities in antiretroviral treatment among HIV-infected pregnant Medicaid enrollees, 2005–2007. Am J Public Health.

[71] Beer L, Bradley H, Mattson CL, Johnson CH, Hoots B, Shouse RL (2016). Trends in racial and ethnic disparities in antiretroviral therapy prescription and viral suppression in the United States, 2009–2013. J Acquir Immune Defic Syndr.

[72] Agénor M, Pérez AE, Koma JW, Abrams JA, McGregor AJ, Ojikutu BO (2019). Sexual orientation identity, race/ethnicity, and lifetime HIV testing in a national probability sample of US women and men: an intersectional approach. LGBT Health.

[73] McCree DH, Beer L, Crim SM, Kota KK, Baugher A, Jeffries WL et al. Intersectional discrimination in HIV healthcare settings among persons with diagnosed HIV in the United States, Medical Monitoring Project, 2018–2019. AIDS Behav. 2023:1–9. 10.1007/s10461-023-04076-2 37166687

[74] Barrington C, Davis DA, Villa-Torres L, Carcano J, Hightow-Weidman L (2021). Intersectionalities and the HIV continuum of care among gay latino men living with HIV in North Carolina. Ethn Health.

[75] Algarin AB, Zhou Z, Cook CL, Cook RL, Ibañez GE (2019). Age, sex, race, ethnicity, sexual orientation: intersectionality of marginalized-group identities and enacted HIV-related stigma among people living with HIV in Florida. AIDS Behav.

[76] Jaworsky D, Logie CH, Wagner AC, Conway T, Kaida A, de Pokomandy A (2018). Geographic differences in the experiences of HIV-related stigma for women living with HIV in northern and rural communities of Ontario, Canada. Rural Remote Health.

[77] McClarty LM, Blanchard JF, Becker ML (2021). Leaving no one behind? An equity analysis of the HIV care cascade among a cohort of people living with HIV in Manitoba, Canada. BMC Public Health.

[78] Konkor I, Lawson ES, Antabe R, McIntosh MD, Husbands W, Wong J et al. An intersectional approach to HIV vulnerabilities and testing among heterosexual African Caribbean and Black Men in London, Ontario: results from the weSpeak study. Augmentative and alternative communication (Baltimore, Md: 1985). 2020;7(6):1140-9. 10.1007/s40615-020-00737-3 32212106

[79] Asiedu GB, Myers-Bowman KS (2014). Gender differences in the experiences of HIV/AIDS-related stigma: a qualitative study in Ghana. Health Care Women Int.

[80] Astawesegn FH, Conroy E, Mannan H, Stulz V (2022). Measuring socioeconomic inequalities in prenatal HIV test service uptake for prevention of mother to child transmission of HIV in East Africa: a decomposition analysis. PLoS ONE.

[81] Ataro Z, Mengesha MM, Abrham A, Digaffe T (2020). Gender differences in Perceived Stigma and coping strategies among people living with HIV/AIDS at Jugal Hospital, Harar, Ethiopia. Psychol Res Behav Manag.

[82] Chirwa GC (2019). Socio-economic inequality in comprehensive knowledge about HIV in Malawi. Malawi Med J.

[83] Faust L, Yaya S, Ekholuenetale M (2017). Wealth inequality as a predictor of HIV-related knowledge in Nigeria. BMJ Global Health.

[84] Geary C, Parker W, Rogers S, Haney E, Njihia C, Haile A (2014). Gender differences in HIV disclosure, stigma, and perceptions of health. AIDS Care.

[85] Girum T, Wasie A, Worku A (2018). Trend of HIV/AIDS for the last 26 years and predicting achievement of the 90-90-90 HIV prevention targets by 2020 in Ethiopia: a time series analysis. BMC Infect Dis.

[86] Hamidouche M, Ante-Testard PA, Baggaley R, Temime L, Jean K (2022). Monitoring socioeconomic inequalities across HIV knowledge, attitudes, behaviours and prevention in 18 sub-saharan African countries. AIDS.

[87] Mori M, Adland E, Paioni P, Swordy A, Mori L, Laker L (2015). Sex differences in antiretroviral therapy initiation in Pediatric HIV infection. PLoS ONE.

[88] Moyo S, Young PW, Gouws E, Naidoo I, Wamicwe J, Mukui I (2018). Equity of antiretroviral treatment use in high HIV burden countries: analyses of data from nationally-representative surveys in Kenya and South Africa. PLoS ONE.

[89] Mudingayi A, Lutala P, Mupenda B (2011). HIV knowledge and sexual risk behavior among street adolescents in rehabilitation centres in Kinshasa; DRC: gender differences. Pan Afr Med J.

[90] Mugoya GC, Ernst K (2014). Gender differences in HIV-related stigma in Kenya. AIDS Care.

[91] Ntata PR, Muula AS, Siziya S, Kayambazinthu EE (2008). Gender differences in university students’ HIV/AIDS-related knowledge and sexual behaviours in Malawi: a pilot study. Sahara j.

[92] Rohleder P, Swartz L, Kalichman SC, Simbayi LC. HIV/AIDS in South Africa 25 years on: psychosocial perspectives. 2009:1–393.

[93] Yao J, Agadjanian V, Murray AT (2014). Spatial and social inequities in HIV testing utilization in the context of rapid scale-up of HIV/AIDS services in rural Mozambique. Health Place.

[94] Kim SW, Skordis-Worrall J, Haghparast-Bidgoli H, Pulkki-Brannstrom AM (2016). Socio-economic inequity in HIV testing in Malawi. Global Health Action.

[95] Ante-Testard PA, Benmarhnia T, Bekelynck A, Baggaley R, Ouattara E, Temime L (2020). Temporal trends in socioeconomic inequalities in HIV testing: an analysis of cross-sectional surveys from 16 sub-saharan African countries Pearl. Lancet Global Health.

[96] Chipanta D, Amo-Agyei S, Giovenco D, Estill J, Keiser O (2022). Socioeconomic inequalities in the 90-90-90 target, among people living with HIV in 12 sub-saharan African countries—implications for achieving the 95-95-95 target—analysis of population-based surveys. Eclinicalmedicine.

[97] Ngandu NK, Van Malderen C, Goga A, Speybroeck N. Wealth-related inequality in early uptake of HIV testing among pregnant women: an analysis of data from a national cross-sectional survey, South Africa. Bmj Open. 2017;7(7). 10.1136/bmjopen-2016-013362 PMC5577866 28706083

[98] Arifin H, Ibrahim K, Rahayuwati L, Herliani YK, Kurniawati Y, Pradipta RO (2022). HIV-related knowledge, information, and their contribution to stigmatization attitudes among females aged 15–24 years: regional disparities in Indonesia. BMC Public Health.

[99] Atteraya M, Kimm H, Song IH (2015). Caste- and ethnicity-based inequalities in HIV/AIDS-related knowledge gap: a case of Nepal. Health Soc Work.

[100] Jesmin SS, Rahman M (2018). Social inequalities and the context of vulnerabilities: HIV/AIDS awareness and prevention knowledge among married women. Health Care Women Int.

[101] Pannetier J, Lelièvre E, Le Cœur S (2016). HIV-related stigma experiences: understanding gender disparities in Thailand. AIDS Care.

[102] Chu D-T, Vo H-L, Tran D-K, Nguyen Si Anh H, Bao Hoang L, Tran Nhu P (2019). Socioeconomic inequalities in the HIV testing during antenatal care in Vietnamese women. Int J Environ Res Public Health.

[103] Sharma SK, Vishwakarma D. Socioeconomic inequalities in the HIV testing during antenatal care: evidence from Indian demographic health survey, 2015–16. BMC Public Health. 2022;22(1). 10.1186/s12889-022-13392-6 PMC9107761 35570285

[104] Chakrapani V, Gulfam FR, Arumugam V, Aher A, Shaikh S, Prasad R et al. Intersectional stigma and gender non-affirmation hinder HIV care engagement among transgender women living with HIV in India. AIDS Care. 2022:1–9. 10.1080/09540121.2022.2099511 PMC9834431 35819879

[105] Elliott L, Parida SK, Gruer L (1992). Differences in HIV-related knowledge, and attitudes between Caucasian and Asian men in Glasgow. AIDS Care.

[106] Taşci S, Başer M, Mucuk S, Bayat M, Zincir H, Sungur G (2008). Erciyes University students’ knowledge about AIDS: differences between students of natural and social science. Behav Med.

[107] van Melle A, Parriault MC, Basurko C, Jolivet A, Flamand C, Pigeon P (2015). Knowledge, attitudes, behaviors, and practices differences regarding HIV in populations living along the Maroni river: particularities of operational interest for Amerindian and Maroon populations. AIDS Care.

[108] Zhussupov B, McNutt LA, Gilbert L, Terlikbayeva A, El-Bassel N (2015). Migrant workers in Kazakhstan: gender differences in HIV Knowledge and sexual risk behaviors. AIDS Behav.

[109] Laut K, Shepherd L, Radoi R, Karpov I, Parczewski M, Mussini C (2018). Persistent disparities in antiretroviral treatment (ART) coverage and virological suppression across Europe, 2004 to 2015. Eurosurveillance.

[110] Li X, Lin C, Gao Z, Stanton B, Fang X, Yin Q (2004). HIV/AIDS knowledge and the implications for health promotion programs among Chinese college students: geographic, gender and age differences. Health Promot Int.

[111] Loutfy MR, Logie CH, Zhang Y, Blitz SL, Margolese SL, Tharao WE (2012). Gender and ethnicity differences in HIV-related stigma experienced by people living with HIV in Ontario, Canada. PLoS ONE.

[112] Zhan J, Fu G, Wu L, Pan M, Yang Y, Chen Z (2021). Inequities in the utilization of HIV counseling and testing services among undergraduates in mainland China. BMC Public Health.

[113] Gutiérrez JP, Trossero A (2021). Socioeconomic inequalities in HIV knowledge, HIV testing, and condom use among adolescent and young women in Latin America and the Caribbean. Rev Panam Salud Publica.

[114] Yang F, Li Z, Subramanian SV, Lu C (2021). Assessment of Knowledge of HIV/AIDS and Association with socioeconomic disparities among Young women in low- and Middle-Income Countries, 2003 to 2018. JAMA Netw Open.

[115] Ghasemi E, Rajabi F, Negarandeh R, Vedadhir A, Majdzadeh R (2022). HIV, migration, gender, and drug addiction: a qualitative study of intersectional stigma towards Afghan immigrants in Iran. Health Soc Care Community.

[116] Auld AF, Shiraishi RW, Mbofana F, Couto A, Fetogang EB, El-Halabi S (2015). Lower levels of antiretroviral therapy enrollment among men with HIV compared with women—12 countries, 2002–2013. Morb Mortal Wkly Rep.

[117] Larose A, Moore S, Harper S, Lynch J (2011). Global income-related inequalities in HIV testing. J Public Health.

[118] Kerrigan D, Vazzano A, Bertoni N, Malta M, Bastos FI (2017). Stigma, discrimination and HIV outcomes among people living with HIV in Rio De Janeiro, Brazil: the intersection of multiple social inequalities. Glob Public Health.

[119] Rohleder P, Eide AH, Swartz L, Ranchod C, Schneider M, Schür C (2012). Gender differences in HIV knowledge and unsafe sexual behaviours among disabled people in South Africa. Disabil Rehabil.

[120] Garofalo R, Gayles T, Bottone PD, Ryan D, Kuhns LM, Mustanski B (2015). Racial/Ethnic difference in HIV-related knowledge among young men who have sex with men and their Association with condom errors. Health Educ J.

[121] Elliott L, Parida S, Gruer L (1992). Differences in HIV-related knowledge and attitudes between Caucasian and ‘Asian’men in Glasgow. AIDS Care.

[122] Ghasemi E, Rajabi F, Negarandeh R, Vedadhir A, Majdzadeh R. HIV, migration, gender, and drug addiction: A qualitative study of intersectional stigma towards Afghan immigrants in Iran. Health & social care in the community. 2021. 10.1111/hsc.13622 34725886

[123] Ante-Testard PA, Benmarhnia T, Bekelynck A, Baggaley R, Ouattara E, Temime L (2020). Temporal trends in socioeconomic inequalities in HIV testing: an analysis of cross-sectional surveys from 16 sub-saharan African countries. Lancet Glob Health.

[124] Fleishman JA, Yehia BR, Moore RD, Gebo KA, Agwu AL (2012). Disparities in receipt of antiretroviral therapy among HIV-infected adults (2002–2008). Med Care.

[125] McNaghten A, Hanson DL, Dworkin MS, Jones JL, Group AASoHD (2003). Differences in prescription of antiretroviral therapy in a large cohort of HIV-infected patients. JAIDS J Acquir Immune Defic Syndr.

[126] Girum T, Wasie A, Lentiro K, Muktar E, Shumbej T, Difer M (2018). Gender disparity in epidemiological trend of HIV/AIDS infection and treatment in Ethiopia. Arch Public Health.

[127] Maulsby CH, Ratnayake A, Hesson D, Mugavero MJ, Latkin CA (2020). A scoping review of employment and HIV. AIDS Behav.

[128] Levi J, Pozniak A, Heath K, Hill A. The impact of HIV prevalence, conflict, corruption, and GDP/capita on treatment cascades: data from 137 countries. Elsevier; 2018:80–90. 10.1016/S2055-6640(20)30249-1 PMC5892682 29682299

[129] Salunke S, Lal DK (2017). Multisectoral approach for promoting public health. Indian J Public Health.

[130] Burris S (2011). Law in a social determinants strategy: a public health law research perspective. Public Health Rep.

[131] Coates TJ, Richter L, Caceres C (2008). Behavioural strategies to reduce HIV transmission: how to make them work better. Lancet.

[132] Geng EH, Nash D, Phanuphak N, Green K, Solomon S, Grimsrud A (2022). The question of the question: impactful implementation science to address the HIV epidemic. J Int AIDS Soc.

[133] Gesesew HA, Tesfay Gebremedhin A, Demissie TD, Kerie MW, Sudhakar M, Mwanri L (2017). Significant association between perceived HIV related stigma and late presentation for HIV/AIDS care in low and middle-income countries: a systematic review and meta-analysis. PLoS ONE.

[134] United Nations. Transforming our world: The 2030 agenda for sustainable development. Resolution adopted by the General Assembly on 25 September 2015. A/RES/70/1. UN General Assembly, Seventieth Session. Agenda items 15 and 116. New York: United Nations; 2015b.

[135] Ortiz-Ospina E, Roser M. Economic inequality by gender. Our World in Data; 2018.

[136] Department SR. Educational attainment worldwide in 2020, by gender and level 2019 [ https://www.statista.com/statistics/1212278/education-gender-gap-worldwide-by-level/#:~:text=Educational attainment worldwide 2020%2C by gender and level&text=According to the Global Gender,males had attained tertiary education

[137] Boag-Munroe G, Evangelou M (2012). From hard to reach to how to reach: a systematic review of the literature on hard-to-reach families. Res Papers Educ.

[138] Gizaw Z, Astale T, Kassie GM (2022). What improves access to primary healthcare services in rural communities? A systematic review. BMC Prim Care.

[139] Tetteh JK, Frimpong JB, Budu E, Adu C, Mohammed A, Ahinkorah BO (2022). Comprehensive HIV/AIDS knowledge and HIV testing among men in sub-saharan Africa: a multilevel modelling. J Biosoc Sci.

[140] Okumu E, Jolly DH, Alston LM, Eley NT, Laws M, MacQueen KM (2017). Relationship between human immunodeficiency virus (HIV) knowledge, HIV-related stigma, and HIV testing among young black adults in a southeastern city. Front Public Health.

[141] Knettel BA, Fernandez KM, Wanda L, Amiri I, Cassiello-Robbins C, Watt MH (2021). The role of community health workers in HIV care engagement: a qualitative study of stakeholder perspectives in Tanzania. J Association Nurses AIDS Care: JANAC.

[142] Hayanga B, Stafford M, Bécares L (2021). Ethnic inequalities in healthcare use and care quality among people with multiple long-term health conditions living in the United Kingdom: a systematic review and narrative synthesis. Int J Environ Res Public Health.

[143] Arora AK, Ortiz-Paredes D, Engler K, Lessard D, Mate KK, Rodriguez-Cruz A (2021). Barriers and facilitators affecting the HIV care cascade for migrant people living with HIV in organization for economic co-operation and development countries: a systematic mixed studies review. AIDS Patient Care STDs.

[144] Joo JY, Liu MF (2021). Culturally tailored interventions for ethnic minorities: a scoping review. Nurs Open.

[145] Armaou M, Araviaki E, Musikanski L (2020). eHealth and mHealth interventions for ethnic minority and historically underserved populations in developed countries: an umbrella review. Int J Community Well-Being.

[146] Anderson LM, Adeney KL, Shinn C, Safranek S, Buckner-Brown J, Krause LK. Community coalition‐driven interventions to reduce health disparities among racial and ethnic minority populations. Cochrane Database Syst Reviews. 2015;(6). 10.1002/14651858.CD009905.pub2 PMC10656573 26075988

[147] Cutilli CC. Health literacy, health disparities and sources of health information in US older adults. Duquesne University; 2015.

[148] Central Statistical Agency [Ethiopia] and ICF International (2017). Ethiopia Demographic and Health Survey 2016.

[149] Müllerschön J, Koschollek C, Santos-Hövener C, Kuehne A, Müller-Nordhorn J, Bremer V. Impact of health insurance status among migrants from Sub-Saharan Africa on access to health care and HIV testing in Germany: a participatory cross-sectional survey. BMC Int Health Hum Rights. 2019;19(1). 10.1186/s12914-019-0189-3 PMC6399910 30832665

[150] Bauer GR (2014). Incorporating intersectionality theory into population health research methodology: challenges and the potential to advance health equity. Soc Sci Med.

[151] Bowleg L (2012). The problem with the phrase women and minorities: intersectionality—an important theoretical framework for public health. Am J Public Health.

[152] Lacombe-Duncan A (2016). An intersectional perspective on Access to HIV-Related Healthcare for Transgender Women. Transgender Health.

[153] Abubakari GMR, Owusu-Dampare F, Ogunbajo A, Gyasi J, Adu M, Appiah P (2021). HIV Education, Empathy, and empowerment (HIVE3): a peer support intervention for reducing Intersectional Stigma as a barrier to HIV Testing among men who have sex with men in Ghana. Int J Environ Res Public Health.

[154] World Health Organization. Everybody’s business -- strengthening health systems to improve health outcomes:WHO’s framework for action Geneva, Switzerland: World Health Organization. 2007 [ https://apps.who.int/iris/handle/10665/43918

