# Supplementary material for: Explaining inequity in knowledge, attitude, and services related to HIV/AIDS: a systematic review
Source: BMC Public Health. 2024 Jul 8;24:1815. doi: 10.1186/s12889-024-19329-5 (PMC11229290; doi:10.1186/s12889-024-19329-5)
Supplement: Supplementary file 1 — Supplementary Material 1: sT1: Search strategy [file 12889_2024_19329_MOESM1_ESM.docx]

| Main terms | sT1: Search strategy updated on May 29, 2023 | Total accessed |
| --- | --- | --- |
| PubMed | ("Attitude"[Mesh] OR "Health Knowledge, Attitudes, Practice"[Mesh] OR Knowledge[Title] OR attitude* [Title] OR "Social Stigma"[Mesh] OR stigma[Title] OR discrimination[Title] OR "test*" [Title/Abstract] OR "antiretroviral therapy, highly active" [MeSH] OR "anti-retroviral agents"[MeSH] OR "highly active antiretroviral therapy" [title/abstract] OR HAART [title/abstract] OR ARV [title/abstract] OR ARVs [title/abstract] OR "anti-retroviral agents" [title/abstract] OR antiretroviral [title/abstract] OR anti-retroviral [title/abstract]) AND (HIV Infection[MeSH] OR HIV[MeSH] OR hiv[tiab] OR hiv infect*[tiab] OR human immunodeficiency virus[tiab]OR "human immune deficiency virus" [tiab] OR human immuno-deficiency virus[tiab] OR "human immune-deficiency virus" [tiab] OR "acquired immunodeficiency syndrome" [title/abstract] OR aids [title/abstract] OR hiv [title/abstract] OR "human immunodeficiency virus" [title/abstract] OR "HIV infections" [title/abstract] OR HIV/AIDS [Title/Abstract] OR "human immunodeficiency virus/acquired immunodeficiency syndrome"[Title/Abstract]) AND (equit*[title/abstract] OR inequit* [title/abstract] OR equalit* [title/abstract] OR equal [title] OR inequalit* [title/abstract] OR unequal[title/abstract] OR disparit* [title/abstract] OR differenc* [title]): Filters applied: English | 1,837 |
| EMBASE | ('attitude'/exp OR 'attitude' OR 'knowledge'/exp OR 'knowledge' OR 'stigma'/exp OR 'stigma' OR 'discrimination'/exp OR 'discrimination' OR 'highly active antiretroviral therapy'/exp OR 'highly active antiretroviral therapy' OR 'antiretroviral therapy'/exp OR 'antiretroviral therapy' OR 'antiretrovirus agent'/exp OR 'antiretrovirus agent' OR 'test'/exp OR test) AND ('human immunodeficiency virus infection'/exp OR 'human immunodeficiency virus'/exp OR 'acquired immune deficiency syndrome'/exp OR 'hiv aids'/exp) AND ('equity'/exp OR 'health disparity'/exp OR 'diversity, equity and inclusion'/exp OR 'inequality'/exp OR 'disparity'/exp) AND [english]/lim | 660 |
| Web of Science | ((TI=(knowledge) OR TI=(attitude) OR TI=(stigma) OR TI=(discrimination) OR TI=(test) OR TI=("HIV test") OR TI=("Highly active antiretroviral therapy" ) OR TI=(HAART) OR TI=("antiretroviral therapy") OR TI=(ART) OR TI=(Treatment) OR TI=(ARV) OR TI=(arts) OR TI=("Anti-Retroviral Agents" ) OR TI=(antiretroviral) OR TI=(antiviral) OR TI=(therapy)) AND (TI=("Acquired immunodeficiency syndrome") OR TI=(AIDS) OR TI=(HIV) OR TI=("Human immunodeficiency virus" ) OR TI=("HIV infections" ) OR TI=(hiv/aids ) OR TI=("human immunodeficiency virus/acquired immunodeficiency syndrome" )) AND (TI=(equit*) OR TI=(inequit*) OR TI=(equalit*) OR TI=(equal) OR TI=(unequal) OR TI=(inequalit*) OR TI=(dispart*) OR TI=(difference)))  Refined by: Languages: English | 516 |
| Scopus | ( TITLE-ABS-KEY ( knowledge  OR  attitude  OR  stigma  OR  discrimination  OR  test  OR  "Highly active antiretroviral therapy"  OR  haart  OR  "Antiretroviral therapy"  OR  art  OR  treatment  OR  arv  OR  arvs  OR  "Anti-Retroviral Agents"  OR  antiretroviral  OR  antiviral  OR  therapy ) )  AND  ( TITLE-ABS-KEY ( "Acquired immunodeficiency syndrome"  OR  aids  OR  hiv  OR  "Human immunodeficiency virus"  OR  "HIV infection"  OR  hiv/aids  OR  "human immunodeficiency virus/acquired immunodeficiency syndrome" ) )  AND  ( TITLE-ABS-KEY ( equit*  OR  inequit*  OR  equalit*  OR  equal  OR  unequal  OR  inequalit*  OR  disparit* ) )  AND  ( LIMIT-TO ( DOCTYPE ,  "ar" ) )  AND  ( LIMIT-TO ( LANGUAGE ,  "English" ) ) | 2933 |
| Google Scholar | Hand searching using title | 84 |
