# Supplementary material for: Explaining inequity in knowledge, attitude, and services related to HIV/AIDS: a systematic review
Source: BMC Public Health. 2024 Jul 8;24:1815. doi: 10.1186/s12889-024-19329-5 (PMC11229290; doi:10.1186/s12889-024-19329-5)
Supplement: Supplementary file 2 — Supplementary Material 2: sT2: Risk of bias assessment of included articles [file 12889_2024_19329_MOESM2_ESM.docx]

sT2: Risk of bias assessment of included articles

| Author/Year | Quality indicators for quantitative studies | | | | | | | | | | |
| --- | --- | --- | --- | --- | --- | --- | --- | --- | --- | --- | --- |
|  | External validity | | | | Internal validity | | | | | | |
|  | 1. Target population a close representation? | 2. Sampling frame a true or close representation? | 3. Random selection? Or a census undertaken? | 4. Likelihood of nonresponse bias minimal? | 5. Data collected directly from the subjects? | 6. Case definition? | 7. Study instrument validity and reliability? | 8. Same mode for all subjects? | 9. Length of the shortest prevalence period appropriate? | 10. Numerators and denominators appropriate? | Overall risky of bias |
| Agwu AL et al/2011 | Yes | Yes | Yes | No | Yes | Yes | Yes | Yes | Yes | Yes | Low |
| Arifin H et al/2022 | Yes | Yes | Yes | No | Yes | Yes | Yes | Yes | Yes | Yes | Low |
| Arnold M et al/2009 | Yes | Yes | Yes | Yes | Yes | Yes | Yes | Yes | Yes | Yes | Low |
| Astawesegn FH et al/2022 | Yes | Yes | Yes | Yes | Yes | Yes | Yes | Yes | Yes | Yes | Low |
| Ataro Z et al/2020 | Yes | Yes | No | Yes | Yes | Yes | Yes | Yes | Yes | Yes | Low |
| Atteraya M et al/2015 | Yes | Yes | Yes | No | Yes | No | No | Yes | Yes | Yes | Moderate |
| Behel SK et al/2008 | Yes | No | No | Yes | Yes | No | No | Yes | Yes | Yes | Moderate |
| Brown LK et al/1990 | No | No | Yes | No | Yes | Yes | Yes | Yes | Yes | Yes | Moderate |
| Burlew AK/2007 | No | Yes | Yes | Yes | Yes | Yes | Yes | Yes | Yes | Yes | Low |
| Chirawa 2019 | Yes | Yes | Yes | No | Yes | Yes | Yes | Yes | Yes | Yes | Low |
| Ebrahim SH et al/ 2004 | Yes | No | No | No | Yes | Yes | Yes | Yes | Yes | Yes | Moderate |
| Elliott L et al/1992 | No | No | No | No | Yes | Yes | No | Yes | Yes | Yes | Moderate |
| Faust L et al/2017 | Yes | Yes | Yes | Yes | No | Yes | Yes | Yes | Yes | Yes | Low |
| Fleishman JA et al/2012 | Yes | Yes | No | No | No | Yes | Yes | Yes | Yes | Yes | Moderate |
| Garofalo R et al/2015 | Yes | Yes | No | No | Yes | Yes | Yes | Yes | Yes | Yes | Low |
| Geary C et al/2014 | No | No | No | No | Yes | Yes | Yes | Yes | Yes | Yes | Moderate |
| Gebo AG et al/2005 | Yes | No | Yes | No | No | Yes | Yes | Yes | Yes | Yes | Moderate |
| Girum T et al/2018 | Yes | Yes | Yes | No | No | Yes | Yes | Yes | Yes | Yes | Low |
| Gutiérrez JP & Trossero A. 2021 | Yes | Yes | Yes | No | No | Yes | Yes | Yes | Yes | Yes | Low |
| Guwani JM et al/2004 | Yes | No | Yes | No | Yes | Yes | No | Yes | Yes | Yes | Moderate |
| Hall HI et al/2013 | No | Yes | Yes | No | No | Yes | No | Yes | Yes | Yes | Moderate |
| Hamidouche M et al/2022 | Yes | Yes | Yes | Yes | No | Yes | No | Yes | Yes | Yes | Low |
| Jaworsky D et al/2018 | No | No | No | No | No | Yes | Yes | Yes | Yes | Yes | Moderate |
| Jesmin S.S. & Rahman M/2018 | Yes | Yes | Yes | No | No | Yes | Yes | Yes | No | Yes | Low |
| Kerrigan D et al/2017 | Yes | No | Yes | Yes | Yes | Yes | Yes | Yes | Yes | Yes | Low |
| Landovitz RJ et al/2017 | Yes | Yes | No | No | No | Yes | Yes | Yes | Yes | Yes | Low |
| Lemly DC et al/2009 | No | No | Yes | No | No | Yes | Yes | Yes | Yes | Yes | Moderate |
| Li X et al/2004 | Yes | Yes | No | Yes | Yes | Yes | Yes | Yes | Yes | Yes | Low |
| Lo CC et al/2018 | Yes | No | No | No | No | Yes | Yes | Yes | Yes | Yes | Moderate |
| Loutfy MR et al/2012 | No | No | No | No | No | Yes | Yes | Yes | Yes | Yes | Moderate |
| McNaghten AD et al/2003 | No | No | No | No | No | Yes | Yes | Yes | Yes | Yes | Moderate |
| Metz VE et al/2017 | No | No | Yes | No | Yes | Yes | Yes | Yes | Yes | Yes | Moderate |
| Miller J E 2000 | No | No | Yes | No | Yes | Yes | Yes | Yes | Yes | Yes | Moderate |
| Mori M et al/2015 | Yes | Yes | Yes | Yes | Yes | Yes | Yes | Yes | Yes | Yes | Low |
| Moyo S et al/2018 | Yes | Yes | No | Yes | Yes | Yes | Yes | Yes | Yes | Yes | Low |
| Mudingayi A et al/2011 | No | Yes | No | No | Yes | Yes | Yes | Yes | Yes | Yes | Low |
| Mugoya GCT et al/2014 | Yes | Yes | Yes | Yes | No | Yes | Yes | Yes | Yes | Yes | Low |
| Ntata PRT et al/2008 | Yes | No | No | Yes | Yes | Yes | No | Yes | Yes | Yes | Low |
| Ojikutu B et al/2013 | No | No | Yes | Yes | Yes | Yes | No | Yes | No | Yes | Moderate |
| Pannetier J et al/2016 | No | No | Yes | No | Yes | Yes | Yes | Yes | Yes | Yes | Low |
| Rapkin AJ&Erickson PI/996 | Yes | Yes | Yes | Yes | Yes | Yes | Yes | Yes | Yes | Yes | Low |
| Rohleder P et al/2012 | No | No | No | No | Yes | Yes | Yes | Yes | Yes | Yes | Moderate |
| Tas¸ci S et al/2008 | Yes | Yes | Yes | Yes | Yes | Yes | Yes | Yes | Yes | Yes | Low |
| van Melle A et al/2015 | Yes | Yes | Yes | No | No | Yes | Yes | Yes | No | Yes | Low |
| Waldner LK et al/1999 | No | No | Yes | Yes | Yes | Yes | Yes | Yes | Yes | Yes | Low |
| Yang F et al/2021 | Yes | Yes | Yes | No | No | Yes | Yes | Yes | Yes | Yes | Low |
| Yao J et al/2014 | Yes | Yes | Yes | No | No | Yes | Yes | Yes | Yes | Yes | Low |
| Zhan J et al/2021 | No | No | No | No | Yes | Yes | Yes | Yes | Yes | Yes | Low |
| Zhang S et al/2014 | yes | yes | yes | Yes | No | yes | yes | yes | yes | yes | low |
| Zhang S et al/2013 | Yes | Yes | Yes | No | No | Yes | Yes | Yes | Yes | Yes | Low |
| Zhussupov B et al/2014 | Yes | Yes | Yes | No | Yes | Yes | Yes | Yes | Yes | Yes | Low |
| Larose A et al/2011 | Yes | Yes | Yes | Yes | Yes | Yes | Yes | Yes | Yes | Yes | Low |
| Wabir N et al/2013 | Yes | Yes | Yes | Yes | Yes | Yes | Yes | Yes | Yes | Yes | Low |
| Ngandu NK et al/2017 | Yes | Yes | Yes | No | No | Yes | Yes | Yes | Yes | Yes | Low |
| Kim SW et al/2016 | Yes | Yes | Yes | No | No | Yes | Yes | Yes | Yes | Yes | Low |
| Chu DT et al/2019 | Yes | Yes | Yes | No | No | Yes | Yes | Yes | Yes | Yes | Low |
| Ante-Testard PA et al/2020 | Yes | Yes | Yes | Yes | No | Yes | Yes | Yes | Yes | Yes | Low |
| McClarty LM et al/2021 | Yes | Yes | Yes | Yes | Yes | Yes | Yes | Yes | Yes | Yes | Low |
| Laut K et al/2018 | Yes | yes | Yes | Yes | Yes | Yes | Yes | Yes | Yes | Yes | Low |
| Auld AF et al/2015 | Yes | Yes | Yes | Yes | Yes | Yes | Yes | Yes | Yes | Yes | Low |
| Beer L et al/2016 | Yes | Yes | Yes | No | No | Yes | Yes | Yes | Yes | Yes | Low |
| Sharma SK et al 2022 | Yes | Yes | Yes | Yes | No | Yes | Yes | Yes | Yes | Yes | Low |
| Chipanta D et al/ 2022 | Yes | Yes | Yes | Yes | Yes | Yes | Yes | Yes | Yes | Yes | Low |
| McCree DH et al/2023 | Yes | Yes | Yes | Yes | Yes | Yes | Yes | Yes | Yes | Yes | Low |
| Algarin AB et al/2019 | Yes | Yes | No | Yes | Yes | Yes | Yes | Yes | Yes | Yes | Low |
| [Agénor](https://pubmed.ncbi.nlm.nih.gov/?sort=pubdate&size=200&term=Ag%C3%A9nor+M&cauthor_id=31314667) M et al/2019 | Yes | Yes | Yes | No | Yes | Yes | No | Yes | Yes | Yes | Low |
| Konkor I et al/2020 | Yes | Yes | No | Yes | Yes | Yes | Yes | Yes | Yes | Yes | Low |

| Qualitative study quality indicators | | | | | | | | | | | |
| --- | --- | --- | --- | --- | --- | --- | --- | --- | --- | --- | --- |
| Article | Congruity between the stated philosophical perspective and the research methodology | Congruity between the research methodology and the research question or objective | Congruity between the research methodology and the methods used to collect data | Congruity between the research methodology and the representation and analysis of data | Congruity between the research methodology and the interpretation of results | A statement locating the researcher culturally or theoretically | Addressing researcher’s influence on the research, and vice- versa | Participants, and their voices, adequately represented | Evidence of ethical approval by an appropriate body | Conclusions drawn in the research report flow from the analysis, or interpretation, of the data | Risk of bias |
| Asiedu GB et al/2014 | Yes | Yes | Yes | Yes | Yes | No | No | Yes | Yes | Yes | Low |
| Chakrapani V et al/2023 | Yes | Yes | Yes | Yes | Yes | No | No | Yes | Yes | Yes | Low |
| Ghasemi Eet al/2021 | Yes | Yes | Yes | Yes | Yes | No | Yes | Yes | Yes | Yes | Low |
| Barrington C et al/2021 | Yes | Yes | Yes | Yes | Yes | No | No | Yes | Yes | Yes | Low |
| Rountree MA et al/2016 | Yes | Yes | Yes | Yes | Yes | No | No | Yes | Yes | Yes | Low |
